# An *ex vivo* preliminary investigation into the impact of parameters on tissue welding strength in small intestine mucosa-mucosa end-to-end anastomosis

**DOI:** 10.3389/fbioe.2023.1200239

**Published:** 2023-06-05

**Authors:** Caihui Zhu, Li Yin, Jianzhi Xu, Haotian Liu, Xiaowei Xiang, Hui Zhao, Jian Qiu, Kefu Liu

**Affiliations:** ^1^ Department of Light Sources and Illuminating Engineering, School of Information Science and Technology, Fudan University, Shanghai, China; ^2^ Academy for Engineering and Technology, Fudan University, Shanghai, China

**Keywords:** HFLTW, tissue fusion, burst pressure, mucosa-mucosa, end-to-end, porcine bowel

## Abstract

**Background:** Tissue welding is an electrosurgical technique that can fuse tissue for small intestine anastomosis. However, limited knowledge exists on its application in mucosa-mucosa end-to-end anastomosis. This study investigates the effects of initial compression pressure, out-put power, and duration time on anastomosis strength *ex vivo* in mucosa-mucosa end-to-end anastomosis.

**Methods:** *Ex vivo* porcine bowel segments were used to create 140 mucosa-mucosa end-to-end fusions. Different experimental parameters were employed for fusion, including initial com-pression pressure (50kPa–400 kPa), output power (90W, 110W, and 140W), and fusion time (5, 10, 15, 20 s). The fusion quality was measured by burst pressure and optical microscopes.

**Results:** The best fusion quality was achieved with an initial compressive pressure between 200 and 250 kPa, an output power of 140W, and a fusion time of 15 s. However, an increase in output power and duration time resulted in a wider range of thermal damage. There was no significant difference between the burst pressure at 15 and 20 s (*p* > 0.05). However, a substantial increase in thermal damage was observed with longer fusion times of 15 and 20 s (*p* < 0.05).

**Conclusion:** The best fusion quality for mucosa-mucosa end-to-end anastomosis *ex vivo* is achieved when the initial compressive pressure is between 200 and 250 kPa, the output power is approximately 140W, and the fusion time is approximately 15 s. These findings can serve as a valuable theoretical foundation and technical guidance for conducting animal experiments *in vivo* and subsequent tissue regeneration.

## 1 Introduction

Colorectal cancer is a prevalent and lethal disease worldwide (Gu et al., 2018; Ferlay et al., 2021). The primary treatment method for this disease involves the removal of the diseased intestinal segment and anastomosis of the remaining normal tissue to form a complete structure. Hand-sewn and stapling devices are the two most common methods used for anastomosing the small intestine (Santini et al., 2013). However, these approaches have limitations, such as leaving foreign materials in the body that may cause complications, including anastomotic fistula, bleeding, and inflammation (Neutzling et al., 2012; Luglio and Corcione, 2019; Lee et al., 2021). Some of the foreign materials stay in organs permanently, whether absorbable (sutures) or non-absorbable (titanium staples); some of them are removed by a separate operation or through natural excretion (defecation), such as the NiTi ring (Pan et al., 2020a). With the advancement of endoscopic procedures, there is a growing need for developing new anastomotic devices that meet the requirements of endoscopes.

High-frequency live tissue welding (HFLTW) is a novel tissue regeneration technology based on electrosurgical sealing technology that originated in the 1990s at the E.O. Paton Electric Welding Institute in Ukraine (Patonivk et al., 2013). HFLTW has many advantages over hand-sewn and stapling devices, such as being less time-consuming, less expensive, easier for surgeons to perform, and less inflammatory. However, achieving sufficient strength with minimal thermal damage to the adjacent tissue is critical for maintaining the normal function of the body after surgery (Vladimirova et al., 2014; Tryliskyy et al., 2018).

Mucosa-mucosa and serosa-serous anastomosis are two methods of alimentary tract anastomosis. In 2007, Smulder et al. (Smulders et al., 2007) used the LigaSure Anastomotic Device to execute mucosa-mucosa side-to-side anastomoses in live pigs and compared with suturing as the control group. In 2010, circular clamps were used to execute serosal-serosal end-to-end anastomoses in *ex vivo* porcine small intestine segments (Winter et al., 2010). In 2013, an anastomotic de-vice based on existing LigaSureTM technology was used to conduct mucosa-mucosa side-to-side anastomoses on porcine bowel *ex vivo* (Arya et al., 2013). In 2015–2017, Zhao et al. (Zhao et al., 2015a; Zhao et al., 2015b; Zhao et al., 2017) has done extensive work on serosal-serosal end-to-end anastomosis of porcine intestines. While there is extensive research on serosa-serosal end-to-end anastomosis, there is limited published data on mucosa-mucosa end-to-end anastomosis, which is essential to avoid secondary injury to the body (Smulders et al., 2007; Winter et al., 2010; Arya et al., 2013; Zhao et al., 2015a; Zhao et al., 2015b; Zhao et al., 2017). Therefore, it is crucial to study the parameters affecting the strength of small intestinal mucosa-mucosa end-to-end fusion, providing experimental support for clinical research of intestinal anastomosis without foreign body residue and subsequent tissue regeneration.

Research studies have revealed that the initial compressive pressure (ICP), output power (P), and duration time (T) have a significant impact on the strength of tissue fusion in side-to-side anastomoses and serosal-serosal end-to-end anastomosis (Christian et al., 2008; Lim et al., 2010; Arya et al., 2013; Zhao et al., 2015a). In this study, we employed a power generator (Ukraine) to investigate the variables affecting the strength of small intestine mucosa-mucosa end-to-end anastomosis *ex vivo*. Using burst pressure and histological sections, we assessed the effects of ICPs, Ps, and Ts on the fusion strength. This study provides crucial theoretical knowledge and technical guidance for conducting animal experiments *in vivo*.

## 2 Materials and methods

### 2.1 Preparation of small intestine samples

**FIGURE 1:**
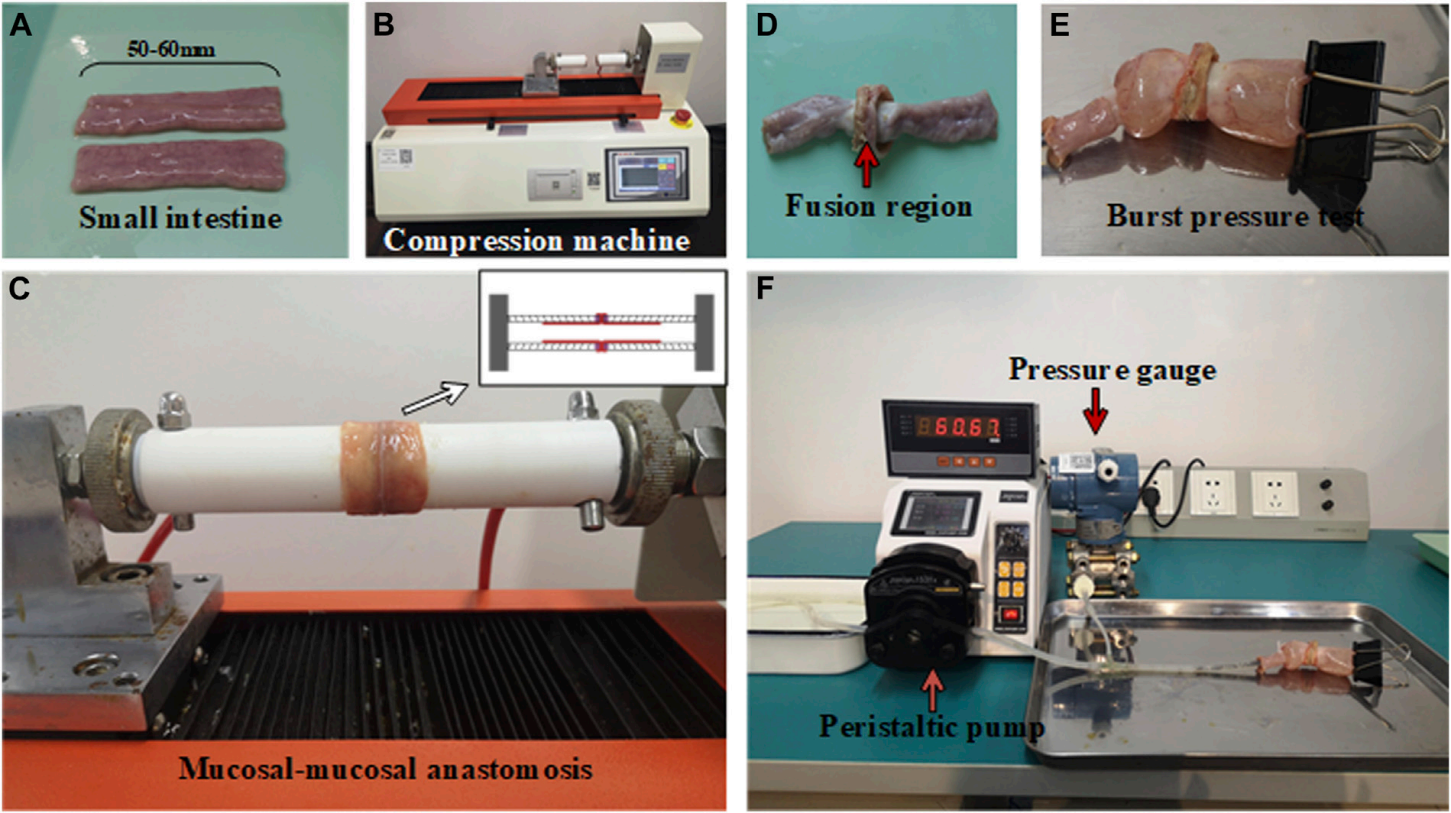
Experimental setups for small intestine fusion and bursting pressure test. **(A)** Small intestine segments. **(B)** Compression machine for HFLTW. **(C)** Mucosa-mucosa anastomosis. **(D)** Fusion region of fused small intestine. **(E)** Burst pressure test for fused small intestine. **(F)** Peristaltic pump for burst pressure measurement.

Fresh small intestines were procured from slaughterhouses and screened before being cut into 50–60 mm segments by a surgeon (as shown in Figure 1A). To minimize experimental variability, we selected small intestines with a thickness ranging from 0.5mm to 1.0 mm. These segments were thoroughly cleaned to remove any fecal matter and then immersed in 0.9% saline solution. They were kept at 0°C–4°C and transported to the laboratory for experimentation within 24 h of harvest.

### 2.2 Experimental settings

The small intestinal fusion apparatus consists of custom-built devices with insulated and heat-resistant hollow round tubes and copper electrodes. The electrodes are mounted on the round tube and are designed to deliver the required pressure to the soft tissue. A compression machine, capable of applying compressive pressure from 0N to 500N (as shown in Figures 1B,C), is used to apply the required pressure. The machine is able to automatically adjust the pressure to maintain a constant pressure during the fusion process. The electrode itself is a metal ring made of copper, which delivers pressure and electricity to the porcine bowel through the electrode. The inner and outer diameters of the electrode measure 13.31 and 23.09 mm, respectively.

To investigate the impact of various experimental parameters on fusion strength, we designed different experimental conditions, including eight different ICP parameters (ranging from 50 to 400 kPa), three P parameters (90, 110, and 140 W), and 4 T parameters (5, 10, 15, and 20 s). The waveform provided by the power generator (EKVZ300) is a square wave with a frequency of 440 khz. Each experimental condition was repeated ten times to ensure reliability. Prior to fusing the porcine bowel, two sections of the bowel were inserted into the hollow round tube and turned outward, with the serosa in close proximity to the outer wall of the tube and the mucosa exposed. The pressure machine then compressed the two pieces of porcine bowel together.

### 2.3 Burst pressure measurement

Burst pressure (BP) refers to the maximum pressure measured by the pressure gauge during infusion. The pressure begins to decrease once the fused anastomotic line begins to leak. To measure BP, a specialized device was used consisting of three components (as shown in Figures 1D–F): a peristaltic pump, a pressure sensor, and a fused small intestine, which were connected via a T-shaped tube. The peristaltic pump is used to push water from the reservoir into the fused intestine at a rate of 5 ml/min. The pressure borne by the pressure sensor is the same as that of the small intestine. The maximum pressure measured by the pressure sensor is considered the BP of the small intestine. In this study, a BP of less than 15.4 mmHg is considered a failure of the welding experiment, while a BP exceeding this value is considered a successful outcome (Kramer and Rentschler, 2018).

### 2.4 Pathological examination

Fused areas were collected and promptly fixed in 10% formalin. The specimens were then dehydrated, made transparent, and immersed in paraffin wax before being cut into 6-um thick transverse slices of the fused area. These slices were then stained with hematoxylin-eosin (H&E) and prepared for viewing. Using a stereo microscope (SZ680; Optics, China), the prepared intestinal slices were visualized and imaged. An experienced histopathologist participated in the study to verify the tissue and cell structure.

### 2.5 Statistical analysis

Statistical analysis was conducted using SPSS software ver.20 (SPSS, IBM, United States). Since the experimental data of BP under different ICP did not follow a normal distribution, nonparametric tests were used. Kruskal–Wallis tests were performed to compare differences in BP between multiple groups. The significance level was set at 0.05 for the Kruskal–Wallis test and was adjusted according to Bonferroni for *post hoc* tests. Additionally, one-way analysis of variance (ANOVA) was used to determine the statistical significance of BP under different Ps and Ts parameters.

## 3 Results

### 3.1 Effects of different ICPs on BP

This study utilized a total of 80 fragments of porcine bowel to conduct BP tests at eight different ICPs when P was 140W and T was 10s. Out of these samples, 35 failed the BP test as the BP was less than 15.4 mmHg. However, at ICPs of 200 kPa and 250kPa, the success rate of BP measurement was 100%, and the minimum BP was higher than 15.4 mmHg. The highest BP recorded was 58.16 mmHg at 250 kPa. BP initially increased and then decreased with an increase in ICP (Table 1).

**TABLE 1:** Burst pressures of small intestines undergoing mucosa-mucosa end-to-end anastomosis induced by HFLTW at varying ICPs.

Compressive pressure (kPa)	Success/total numbers	Burst pressure (mean ± SD) (mmHg)	Highest burst pressure (mmHg)	Burst pressure range (mmHg)
50	2/10	9.40 ± 6.67	20.73	0–20.73
100	2/10	11.30 ± 6.52	20.01	0–20.01
150	8/10	23.40 ± 8.10	33.10	5.89–33.10
200	10/10	27.20 ± 5.29	35.26	17.84–35.26
250	10/10	35.05 ± 10.28	58.16	22.30–58.16
300	7/10	29.70 ± 17.86	49.00	0–49.00
350	4/10	14.60 ± 13.62	40.00	0–40.00
400	2/10	8.10 ± 8.34	23.69	0–23.69
Total number fusion:60				

kPa. Kilopascal, SD., standard deviation, mmHg. mm of mercury

**FIGURE 2:**
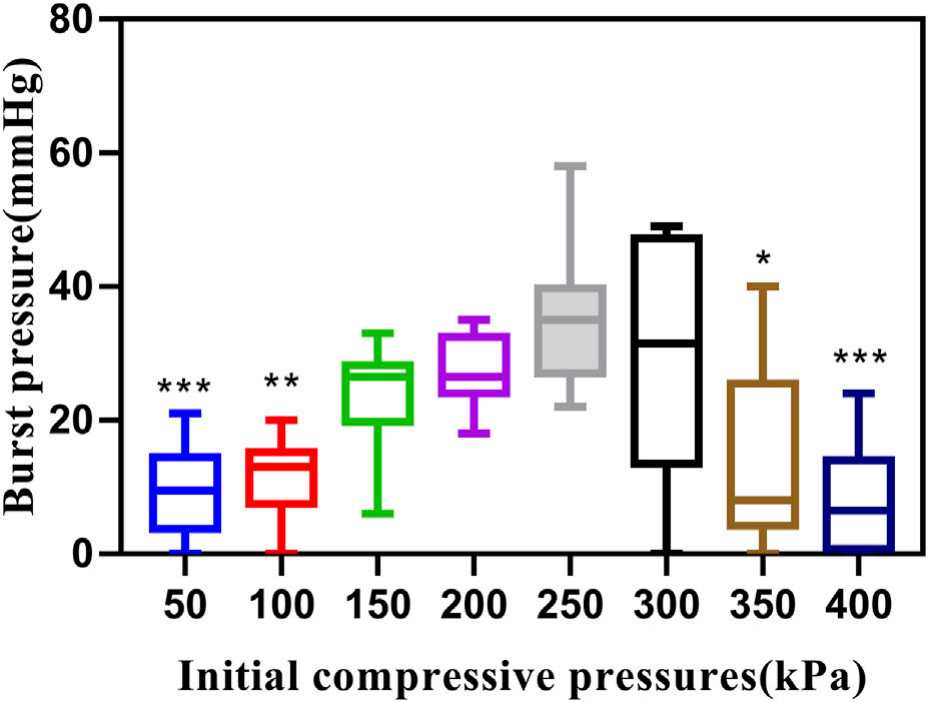
Effects of different ICPs on BP. Graph showing boxplots for BP of small intestines undergoing mucosa-mucosa end-to-end anastomosis induced by HFLTW at varying ICPs (50–400 kPa) where P is 140W and T is 10s. Plots show the medium value (middle line), lower quartile, upper quartile, and data range.

A Kruskal–Wallis test indicated that there was at least one statistically significant difference between these groups (*p* < 0.001). Pair-wise comparisons among the eight levels of ICP groups revealed significant statistical differences in BP measurements between 250kPa and 50 kPa (*p* = 0.001), 100 kPa (*p* = 0.004), 350 kPa (*p* = 0.025), and 400 kPa (*p* < 0.001) (Figure 2). Significant differences in BP were also observed between 200kPa and 50 kPa (*p* = 0.02), 400kPa and 300 kPa (*p* = 0.023), and 400kPa and 200 kPa (*p* = 0.011). However, other groups that were not mentioned in the pair-wise comparisons did not show statistically significant differences. A step change was observed between 100 and 150 kPa, where a higher BP was recorded after a greater pressure than 100 kPa. This occurred because, at ICPs less than 100 kPa such as 50kPa, the gap between tissues was large, which negatively affected the welding quality.

### 3.2 Effects of different Ps on BP

**FIGURE 3:**
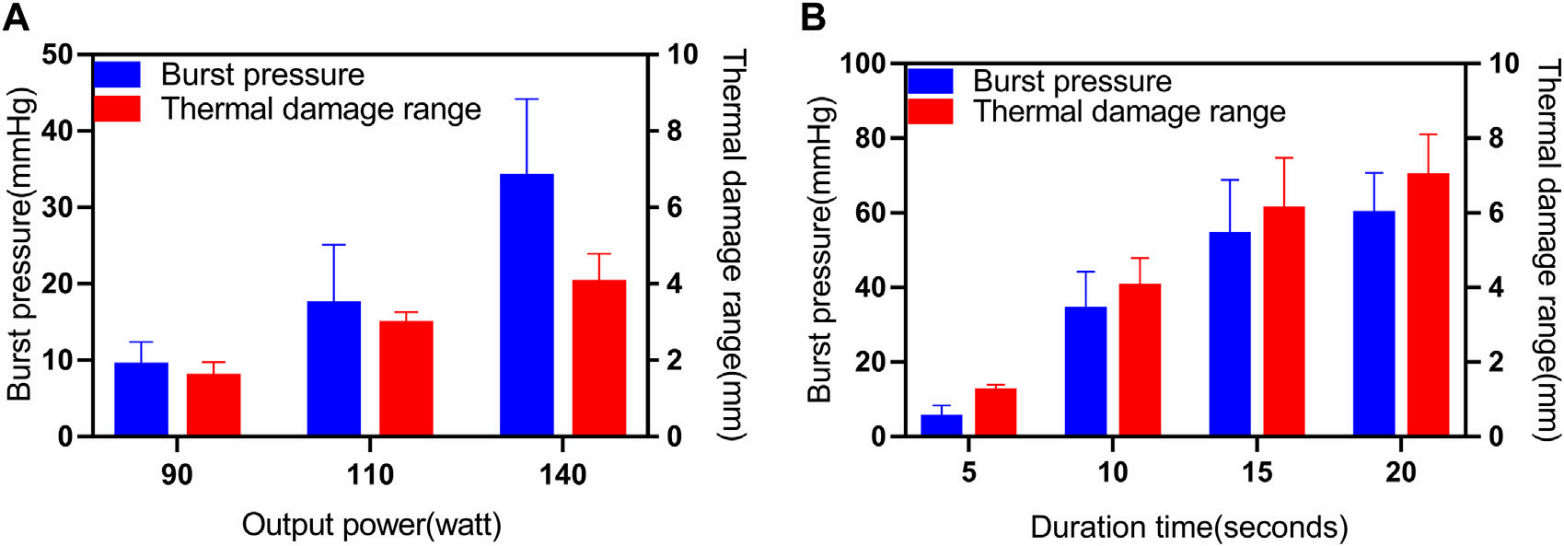
Effects of different parameters (P, T) on BP and thermal damage range. **(A)** Results for the BP and thermal damage range under different Ps (90W, 110W and 140 W), where T is 10s and ICP is 250 kPa. **(B)** Results for the BP and thermal damage range under different Ts (5s, 10s, 15s and 20s), where P is 140W and ICP is 250 kPa.

A total of 60 sections of porcine bowel were fused for BP testing using different power levels and fusion times, as shown in Figure 3 and Table 2. The success rate of BP measurement was 100% at 140w (P3). The highest BP was 52.89 mmHg(P3) and the lowest BP was 5.99 mmHg(P1). The mean BP of P3 (34.08 ± 10.2 mmHg) was higher than that of P1 and P2. A statistically significant differences was observed between BPs of P3(140W) and P1(90W) (34.08 ± 10.2 vs. 9.41 ± 2.97 mmHg, *p* < 0.001), P2(110W) (34.08 ± 10.2 vs.17.36 ± 7.84 mmHg, *p* = 0.0001) (Figure 3A). However, no significant difference was observed between the BPs of P1 (90W) and P2 (110W) (9.41 ± 2.97 vs. 17.36 ± 7.84 mmHg, *p* = 0.068 > 0.05).

**TABLE 2:** Burst pressures of small intestines undergoing mucosa-mucosa end-to-end anastomosis induced by HFLTW at varying Ps and Ts.

Group	Success/total numbers	P(W)	T(s)	Burst pressure (mean ± SD) (mmHg)	Burst pressure range (mmHg)
P1	0/10	90	10	9.41 ± 2.97	5.99–14.00
P2	6/10	110		17.36 ± 7.84	5.85–30.82
P3	10/10	140		34.08 ± 10.2	22.61–52.89
T1	2/10	140	5	5.04 ± 3.18	1.04–9.04
T2	10/10		15	54.92 ± 14.8	33.36–83.75
T3	10/10		20	59.8 ± 10.9	39.47–80.1
Total number fusion:60					

SD, standard deviation, mmHg mm of

### 3.3 Effects of different Ts on BP

A total of 30 sections of porcine bowel were fused for BP testing using 4 different fusion time levels as shown in Figure 3 and Table 2. The success rate of BP measurement was 20% for the T1 group, while it was 100% for the P3, T2, and T3 groups. The highest BP was recorded at the T2 group, which was 83.75 mmHg, whereas the lowest BP was 1.04 mmHg at the T1 group. The mean BP of the T3 group (59.8 ± 10.9 mmHg) was higher than that of the T1, T2, and P3 groups.

Statistically significant differences were observed between the BPs of the T1 group (140W, 5s) and the P3 group (140W, 10s) (*p* < 0.001), T2 group (140W, 15s) (*p* < 0.001), T3 group (140W, 20s) (*p* < 0.001), as well as between the BPs of the P3 group (140W, 10s) and the T2 group (140W, 15s) (*p* = 0.003), and T3 group (140W, 20s) (*p* < 0.001) (Figure 3B). However, no significant difference was observed between the BPs of the T2 group (140W, 15s) and the T3 group (140W, 20s) (*p* > 0.05).

### 3.4 Effects of different parameters on thermal damage range

The results for the thermal damage range at different Ps and Ts were displayed in Figure 3. In this study, the thermal damage range was defined as the distance from the end of the fusion region to the normal tissue, as observed under a microscope. Microscopic examination with H&E staining of unfused (control) porcine bowel revealed normal intact glandular and villous structures in the muscularis mucosa (MM), submucosa (SM), longitudinal muscle (LM), serosa (S), and the boundaries of each area of normal small intestinal tissue were clearly defined (see Supplementary Figure S1). Following tissue welding, the tissue was damaged by heat, and the muscle layer exhibited the most notable changes in microstructure. The cell structure of the mucosa muscle layer in normal intestinal tissue was clearly defined. However, when the small intestine was fused using HFLTW, the nucleus of the mucosa muscle layer in the surrounding tissue of the fusion area appeared fuzzy, and the appearance of some structures in the mucosa muscle layer became “feather shape,” which is an important feature of tissue thermal damage (see Supplementary Figure S2). Significant differences were observed in the thermal damage range between 4 T values and three *p* values (*p* < 0.05).

### 3.5 Histological characteristics

**FIGURE 4:**
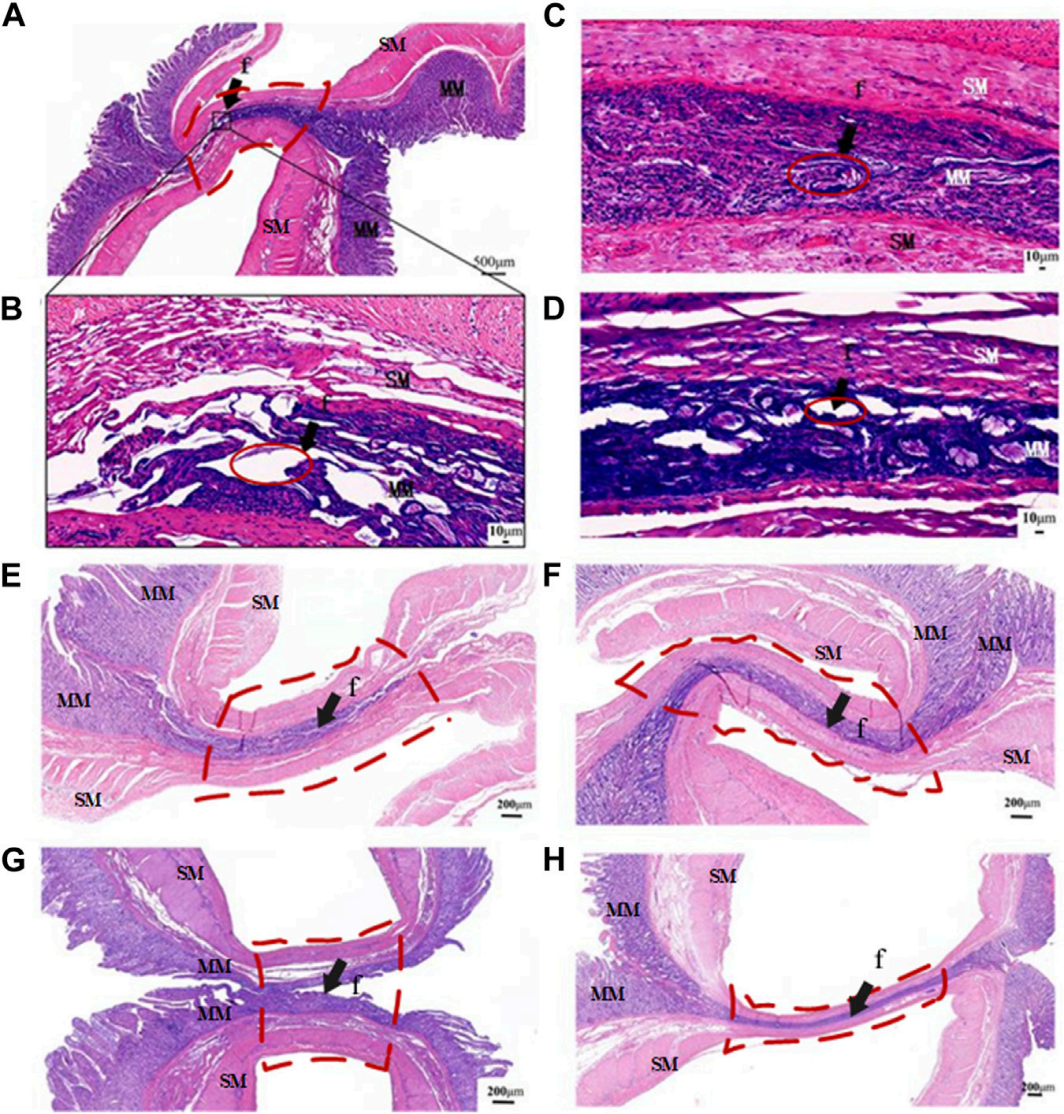
Representative transverse slices of fused area stained with H&E. **(A)** Transverse section of intact fused small bowel following application of 50 kPa. **(B–D)** Transverse section of fused small bowel at ICPs of 50 kPa,100kPa, 250kPa and 350 kPa. **(E–H)** The fusion parameters correspond to P1 (10 s at 90W), P3 (10s and 140W), T1 (5s at 140W) and T2 (15s and 140W), as defined in Table 2. MM, muscularis mucosa; SM, submucosa; f, fusion area (arrows). Red solid line area is the gap at the fusion area, and red dotted line area is the region of interest.

The application of HF LTW with varying ICPs resulted in a variable fusion effect in the small intestine. When an ICP of 50 kPa was applied, large holes were present in the mucosa layer, and obvious gaps could be seen between the two mucous membranes, as shown in Figures 4A,B. As the ICP increased, the pores gradually became smaller. At an ICP of 250kPa, the boundaries of the fusion regions were completely eliminated, and the fusion region became a whole, as shown in Figure 4C. However, at an ICP of 350kPa, the pores in the fusion region reappeared with larger and wider gaps, and the boundary was clearly visible between the two small intestinal tissues, as shown in Figure 4D.

When the output power of 90W was applied for fusing at an ICP of 250 kPa and T of 10s, boundaries and small holes in the fusion area could be seen clearly between the mucous and musculature layers, as shown in Figure 4E. When the power was increased to 140W, the mucosa and mucosa muscle layers of the two small intestinal tissues were fused into a whole, and the tissue boundaries of the fusion area became blurred, with no obvious gaps and holes visible, as shown in Figure 4F.

However, when the welding time was only 5s at an ICP of 250 kPa and power of 140W, HF LTW failed completely, and there was a large gap between the tissues, which separated the two tissues completely, as shown in Figure 4G. In comparison, when the welding time was extended to 15s, the mucous membranes of the two tissues were tightly joined together, and the fusion area became thinner, with no holes visible on the muscularis mucosa, as shown in Figure 4H. Prolongation of welding time can improve welding strength and burst pressure, and the maximum burst pressure can reach 98.09 mmHg. However, with the prolongation of fusion time, the thermal damage range of the surrounding tissues also becomes larger.

## 4 Discussion

HFLTW is a novel surgical anastomosis technique that has proven successful in porcine bowel closure and vascular anastomosis (Christian et al., 2008; Sanchez-De Pedro et al., 2014; Han et al., 2015; Kramer and Rentschler, 2018). This method can perform both serosa-serosa and mucosa-mucosa anastomosis in porcine bowel. Traditional porcine bowel closing staplers were primarily based on mucosa-mucosa anastomosis, while circular staplers were based on serosa-serosa anastomosis (Santini et al., 2013). However, due to the unique structure of the intestinal tract, circular stapler end-to-end anastomosis requires an opening at the other end of the intestinal tract for escape, which can increase the burden of wound healing. In contrast, mucosa-mucosa anastomosis can be performed without causing additional harm to the body (refer to Supplementary Figure S3).

The mechanical mechanism of thermo-fusion involves protein denaturation, cross-linking, and reannealing, which can be influenced by several parameters including ICP, pressure, and temperature (Zhao et al., 2015a). Previous studies have demonstrated that the average BP increases with an increase in ICP before reaching the optimal range (Arya et al., 2013; Zhao et al., 2015b; Zhao et al., 2017). However, beyond this range, an increase in ICP does not result in increased BP (Arya et al., 2013). This is because as ICP increases, the tissue impedance also increases until it reaches its peak value. Further increases in pressure cause a decrease in impedance, leading to a decrease in welding strength and BP (Dodde et al., 2012). Zhao’s study in 2017 found that the highest success rate for BP measurement was achieved with a compression pressure of 171–277 kPa during serosa-serosa anastomosis (Zhao et al., 2017). In the present study, the optimal ICP range was found to be 200–250 kPa, with a 100% success rate for BP measurement. This finding has significant implications for the development of clinical devices. Within the optimal ICP range, the maximum mean BP for mucosa-mucosa end-to-end fusion was 35.05 ± 10.23 mmHg, while the highest mean BPs were 41.0 ± 7.4 mmHg for serum-serosal end-to-end fusion and 27.56 ± 7.51 mmHg for mucosa-mucosa closure, respectively (Winter et al., 2010; Arya et al., 2013). However, to date, no literature has reported the minimum BP required for RF-induced intestinal anastomoses.

ICP is a crucial factor for successful tissue fusion, but to ensure a smooth and effective process, an appropriate energy dose related to power (P) and welding time (T) is also necessary (Winter et al., 2010; Tu et al., 2019). P and T primarily affect the quality of welding by influencing collagen degeneration and cross-linking. Longer welding times or greater power can increase the reducible crosslinks of collagen while reducing non-reducible crosslinks (Su et al., 2014). Pan’s research showed that using low output power could result in an increased risk of anastomotic fistula and wound infection in experimental animals, which could even lead to death (Pan et al., 2020a). Therefore, adjusting the power and welding time to increase the BP can help reduce the risk of such complications. Our observations revealed a significant difference in BP and lower BP in the anastomotic area under light microscopy when P was set at 90W or 110W. Increasing P from 90W to 140W led to a higher BP, resulting in the mucosa layer merging into a whole. However, finding the balance point between P and T is crucial for a successful outcome.

In our study, we observed a significant improvement in anastomosis strength when the fusion time increased from 5s to 10s, which is consistent with findings from other studies (Reyes et al., 2012; Zhao et al., 2015a). However, we found that further increases in fusion time do not enhance anastomosis strength but instead cause damage to the surrounding tissue. Specifically, we found that a fusion time of 20s does not contribute to an increase in anastomosis strength but actually leads to greater thermal damage to the surrounding tissue. In addition, we observed that when the fusion time exceeded 10s, a large amount of water vapor spilled from the anastomosis, causing tissue contraction and potentially leading to anastomotic tear and reduced quality of the anastomosis. Furthermore, we found that the water vapor escaping from tissues in the early stage of the fusion process can cause tissue damage (Floume et al., 2008; Floume et al., 2010). Additionally, the predicted shrinkage of collagen fibrils to one-third of their length when heated (Hormann and Schlebusch, 1971) could also contribute to the splitting of the fusion region. Therefore, it is crucial to minimize the duration of the fusion time while ensuring the quality of the anastomosis.

HFLTW method can effectively ensure a certain level of anastomotic strength when optimal ICPs, Ts, and Ps parameters are utilized. These three parameters are crucial for the research and development of small intestine welding stapling and require the use of two sensors, one for pressure and another for temperature, to accurately monitor the process. The ICP plays a decisive role in the success or failure of the HFLTW process, thus requiring precise control during small intestine welding, This is also the biggest difference between HFLTW and the current cutting methods in electrosurgery. The P and T parameters affect the temperature, with low temperatures failing to achieve proper fusion strength and high temperatures leading to unnecessary thermal damage to surrounding tissues and even damage the anastomotic site. Therefore, monitoring temperature during the fusion process is essential. As the fused tissue induced by HFLTW lacks mechanical support, other materials such as Bio glue (Kramer and Rentschler, 2018), mesenchymal stem cells (Pan et al., 2020b), and platelets can be applied to improve fusion strength and reduce surgical risks. The mechanism of HFLTW differs from that of radiofrequency ablation. Radiofrequency ablation increases cell membrane permeability through irreversible electroporation, which ultimately leads to cell death (Jaber et al., 2016). In contrast, HFLTW achieves instant tissue fusion through the thermal effect of electricity, which denatures collagen (Zhao et al., 2015a). Radiofrequency ablation has a high electric field intensity but a short duration of action (Jiang et al., 2015), whereas HFLTW has a low electric field intensity but a longer duration of action. Radiofrequency ablation and HFLTW are two different instruments used in distinct applications. Radiofrequency ablation is mainly used in the field of tumor treatment, such as in lung cancer and liver cancer (Hester et al., 2014; Jiang et al., 2015; Jaber et al., 2016). Its unique electrical parameters cannot be used for tissue reconstruction On the other hand, HFLTW is primarily used in surgical suturing, such as in the reconstruction of blood vessels and intestines which cannot be used for tumor ablation (Nunvpvanpb et al., 2014; Arya et al., 2015). HFLTW, as a new type of anastomosis technique, is currently undergoing rapid development. There is no device for intestinal mucosa-mucosa end-to-end fusion *in vivo*. Therefore, a large number of *in vivo* and *in vitro* experiments on small intestine anastomoses are necessary to assess postoperative recovery. In this study, a large number of *ex vivo* experiments were done to study the influence of different parameters on the fusion effect of tissues, and these parameters have important reference value for future *in vivo* experiments and Mathematical model calculation. There are two main limitations in this study. The first limitation is that the experiment has not been verified by *in vivo* experiments. The second limitation is that the apparatus involved in the experiment is only suitable for *ex vivo* research, not for *in vivo* surgery. In the future, *ex vivo* and *in vivo* studies should be combined; in addition to this, the sample size should be increased and the amount of clinical data should be increased to make the results more scientific.

## 5 Conclusion

This research examined the impact of three parameters, namely, ICP, P and T, on the strength of small intestine mucosa-mucosa end-to-end anastomosis *ex vivo*. The findings revealed that the optimal fusion quality was achieved when ICP ranged between 200 and 250KPa, P was 140W and T was 15s. Attaining an optimal ICP range is crucial for successful tissue thermo-fusion. P and T influence the fusion quality through energy dose, where an insufficient energy dose will decrease the fusion quality, and an excessive energy dose will cause unwarranted thermal damage to surrounding tissue. As of now, there are no end-to-end thermal fusion devices, and investigating these three parameters using an isolated porcine colon model for end-to-end intestinal anastomosis *in vitro* can have significant clinical implications. Based on the current results of *in vitro* experiments, HFLTW is a promising anastomosis method that may replace existing anastomotic devices in the future. HFLTW has not yet been truly used in clinical practice, and this study is only a preliminary exploration of the control parameters. These findings can provide a basic foundation and reference for future clinical research and mathematical simulation.

## Data Availability

The original contributions presented in the study are included in the article/Supplementary Material, further inquiries can be directed to the corresponding author.
