# Supplementary material for: An ex vivo preliminary investigation into the impact of parameters on tissue welding strength in small intestine mucosa-mucosa end-to-end anastomosis
Source: Front Bioeng Biotechnol. 2023 Jun 5;11:1200239. doi: 10.3389/fbioe.2023.1200239 (PMC10277648; doi:10.3389/fbioe.2023.1200239)
Supplement: Supplementary file 1 [file DataSheet1.docx]

Supplementary Material

An ex vivo preliminary investigation into the impact of parameters on tissue welding strength in small intestine mucosa-mucosa end-to-end anastomosis

Caihui Zhu ^1^, Li Yin ^1^, Jianzhi Xu^1^, Haotian Liu^2^, Xiaowei Xiang^2^, Hui Zhao^1^, Jian Qiu^1^, Kefu Liu^1,^*

*** Correspondence:** Kefu Liu: [kfliu@fudan.edu.cn](mailto:kfliu@fudan.edu.cn)

## Supplementary Figures：


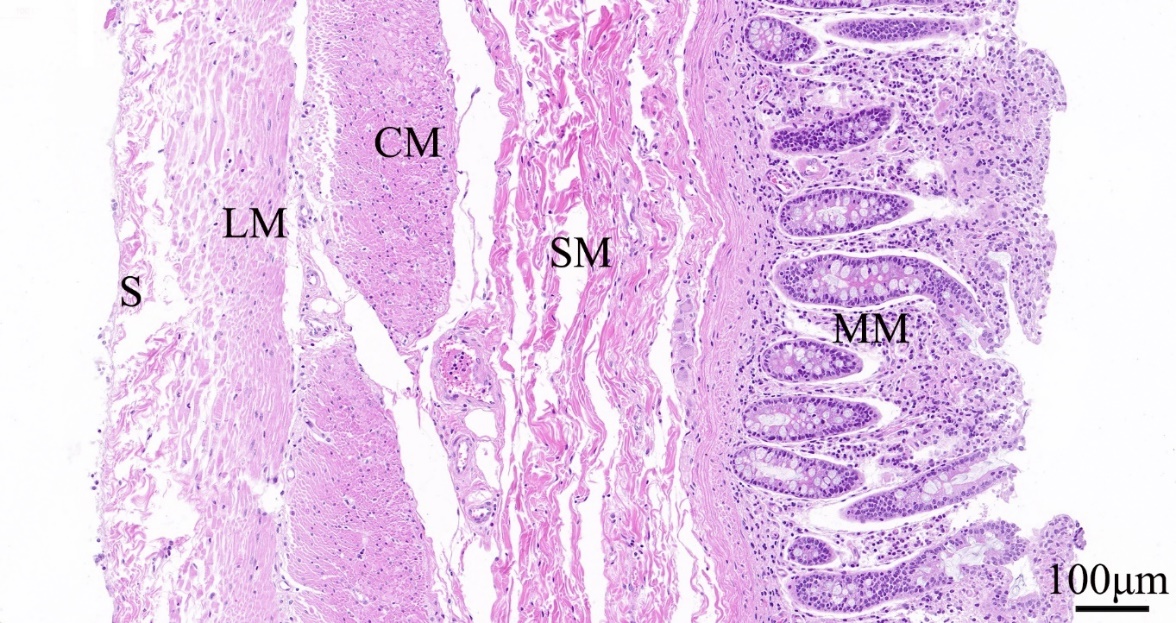


**Supplementary Figure.S1**. Transverse slice and H&E stain of normal small intestine.MM, muscularis mucosa; SM, submucosa; CM, circular muscle; LM, longitudinal muscle; S, serosa.


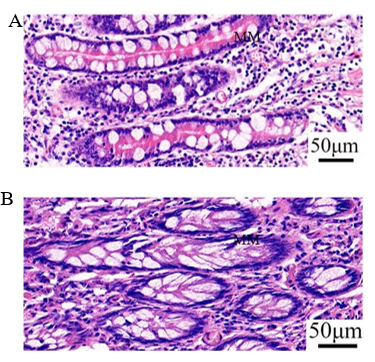


**Supplementary Figure.S2.** A. Mucosa muscularis of normal porcine bowel (H&E stain). B. Injured mucosa muscularis near fusion region (H&E stain).


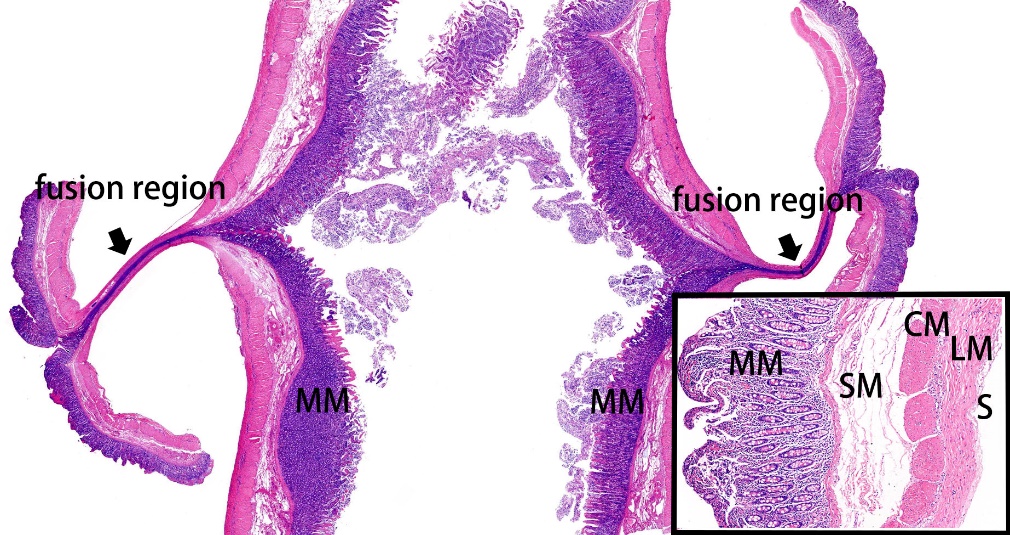


**Supplementary Figure.S3**. Transverse slice and H&E stain (9 magnifications) of porcine bowel undergoing mucosa-mucosa end-to-end anastomosis. Inset shows magnification of unfused area (100 magnifications).MM, muscularis mucosa; SM, submucosa; CM, circular muscle; LM, longitudinal muscle; S, serosa.
